# Fecal Virome Transplantation Confirms Non-Bacterial Components (Virome and Metabolites) Participate in Fecal Microbiota Transplantation-Mediated Growth Performance Enhancement and Intestinal Development in Broilers with Spatial Heterogeneity

**DOI:** 10.3390/microorganisms13081795

**Published:** 2025-07-31

**Authors:** Shuaihu Chen, Tingting Liu, Junyao Chen, Hong Shen, Jungang Wang

**Affiliations:** 1 College of Animal Science and Technology, Shihezi University, Shihezi 832061, China; 17867594001@163.com (S.C.); tingtingliu0212@163.com (T.L.); 18396408986@163.com (J.C.); 2 College of Agriculture, Shihezi University, Shihezi 832061, China

**Keywords:** fecal microbiota transplantation, fecal virome transplantation, intestinal development, virome, metabolites, gut microbial safety

## Abstract

Fecal microbiota transplantation (FMT) promotes growth performance and intestinal development in yellow-feathered broilers, but whether the virome and metabolites contribute to its growth-promoting effect remains unclear. This study removed the microbiota from FMT filtrate using a 0.45 μm filter membrane, retaining the virome and metabolites to perform fecal virome transplantation (FVT), aiming to investigate its regulatory role in broiler growth. Healthy yellow-feathered broilers with high body weights (top 10% of the population) were used as FVT donors. Ninety-six 8-day-old healthy male yellow-feathered broilers (95.67 ± 3.31 g) served as FVT recipients. Recipient chickens were randomly assigned to a control group and an FVT group. The control group was gavaged with 0.5 mL of normal saline daily, while the FVT group was gavaged with 0.5 mL of FVT solution daily. Growth performance, immune and antioxidant capacity, intestinal development and related gene expression, and microbial diversity were measured. The results showed that FVT improved the feed utilization rate of broilers (the feed conversion ratio decreased by 3%; *p* < 0.05), significantly increased jejunal length (21%), villus height (69%), and crypt depth (84%) (*p* < 0.05), and regulated the jejunal barrier: insulin-like growth factor-1 (*IGF-1*) (2.5 times) and Mucin 2 (*MUC2*) (63 times) were significantly upregulated (*p* < 0.05). FVT increased the abundance of beneficial bacteria *Lactobacillales*. However, negative effects were also observed: Immunoglobulin A (IgA), Immunoglobulin G (IgG), Immunoglobulin M (IgM), Interleukin-1 beta (IL-1β), Interleukin-6 (IL-6), Tumor Necrosis Factor-alpha (TNF-α), and Interferon-gamma (IFN-γ) in broilers were significantly upregulated (*p* < 0.05), indicating immune system overactivation. Duodenal barrier-related genes Mucin 2 (*MUC2*), Occludin (*OCLN*), Claudin (*CLDN1*), and metabolism-related genes solute carrier family 5 member 1 (*SLC5A1*) and solute carrier family 7 member 9 (*SLC7A9*) were significantly downregulated (*p* < 0.05). The results of this trial demonstrate that, besides the microbiota, the gut virome and metabolites are also functional components contributing to the growth-promoting effect of FMT. The differential responses in the duodenum and jejunum reveal spatial heterogeneity and dual effects of FVT on the intestine. The negative effects limit the application of FMT/FVT. Identifying the primary functional components of FMT/FVT to develop safe and targeted microbial preparations is one potential solution.

## 1. Introduction

The gut microbiota refers to the microbial communities colonizing the intestinal tract, encompassing viruses, prokaryotes (bacteria and archaea), and eukaryotes (fungi and parasites). These microorganisms regulate host immune function, nutrient absorption, and intestinal mucosal barrier function through metabolites (such as short-chain fatty acids {SCFAs}, virulence factors, etc.) [1], and are thus considered the “second digestive organ” of animals. However, the early-life gut microbiota [2] of livestock and poultry exhibits a simplistic structure and limited digestive capacity [3], making it difficult to compensate for the insufficiency of early physiological digestive function. Consequently, modulating the gut microbiota structure is a crucial strategy for enhancing early growth performance in these animals [4].

Currently, microbial modulation in livestock production primarily relies on probiotic supplementation and antibacterial agents [5,6,7]. However, both approaches exhibit certain limitations: probiotics are frequently affected by host individual differences in colonization rates and functional stability within the complex gut environment [8,9]; antibacterial agents readily lead to enhanced microbial resistance and disrupted microecological balance [10]. Against this background, fecal microbiota transplantation (FMT) demonstrates distinctive advantages. FMT, as a modulation strategy, involves transplanting the entire microbial community from the feces of healthy donor animals into recipient animals [11], thereby reshaping their gut microbial structure to exert multifaceted effects. Compared to mammals, poultry animals face challenges in the vertical transmission of the maternal microbiota to offspring due to their oviparous mode of reproduction [12]. Additionally, their shorter digestive tract and the difficulty in microbial colonization result in an early-life gut microecosystem characterized by low diversity and high plasticity [13]. This inherent characteristic enables FMT to rapidly reconstitute the gut microbial network in chicks and amplify its regulatory effects. Ma et al. revealed that the underlying mechanism may be associated with the microbiota-mediated regulation of the jejunal Th17/Treg cell balance and increased jejunal length in recipient chickens [14]. Liu et al. further proposed that FMT promotes growth by activating the expression of genes related to the growth hormone/insulin-like growth factor-1 (GH/IGF-1) signaling pathway in the liver [15]. Notably, both studies identified beneficial bacteria such as Lactobacillus as the primary functional components mediating these effects.

It is noteworthy that beyond bacteria, other components within the FMT material, including viruses (e.g., bacteriophages), metabolites, active proteins, and microbial products, represent potential functional elements [16]. Research by Podlacha et al. found that lytic bacteriophages could specifically clear Salmonella Enteritidis infection in broilers [17], significantly enhancing systemic antioxidant capacity and intestinal mucosal barrier function, thereby alleviating oxidative stress and inflammatory responses. Saleh et al. reported that dietary supplementation with bacteriophages in Guangxi partridge chickens reduced the feed-to-gain ratio, the diarrhea rate, and mortality, increased intestinal Lactobacillus counts, decreased counts of Escherichia coli and Salmonella, and enhanced immune function [18]. Considering the dominant abundance of bacteriophages in the gut (reaching up to 10^15^ particles per gram) [19] and their regulatory capacity over the intestinal barrier [20], it is suggested that the virome may be a significant functional component of FMT.

Fecal virome transplantation (FVT) can be regarded as a component-specific transplantation derived from FMT, representing an advanced form of this technique [16]. FVT builds upon FMT by employing filtration to remove the bacterial fraction [21], specifically enabling the study of the gut virome and metabolites. Research indicates that FVT exhibits efficacy similar to that of FMT: both can effectively treat Clostridioides difficile infection (CDI) [22], improve gut microbiota composition and intestinal barrier function in broilers [23], and accelerate recovery from lipopolysaccharide (LPS)-induced intestinal inflammation in broilers [24]. However, in contrast to studies demonstrating that FMT from high-body-weight chickens promotes growth performance in chicks, whether high-body-weight chicken FVT exerts an equivalent growth-promoting effect remains largely unexplored.

Therefore, this study aims to investigate the impact of FVT from high-body-weight chickens on the growth performance and intestinal development of chicks, providing a theoretical foundation for subsequent research.

## 2. Materials and Methods

### 2.1. Experimental Design and Broilers Management.

This trial adopted a single-factor, completely randomized design. Twelve 42-day-old healthy male yellow-feathered broilers (with body weights in the top decile of the population) served as donors for fecal virome transplantation (FVT), and ninety-six 8-day-old healthy male yellow-feathered broilers (95.67 ± 3.31 g) were used as FVT recipients. The recipient birds were randomly assigned to two groups: the CON group received daily oral gavage of 0.5 mL physiological saline, and the FVT group was administered a 0.5 mL FVT suspension daily. Each group had six replicates, with eight birds per replicate. After a 14-day experimental period, one bird from each replicate was euthanized for tissue sampling on Day 14. All broilers were housed in vertical cages (2.0 m × 0.9 m × 1.8 m) with continuous ad libitum access to feed and water. Immunization protocols and disinfection procedures were strictly implemented in accordance with commercial farm standards.

### 2.2. Preparation of FVT Suspension

The fecal virome transplantation (FVT) suspension was prepared in accordance with the method described by previous researchers with modifications [21]. Fresh fecal samples collected from donor chickens were homogenized and transported on ice to the laboratory. Sterile physiological saline was added to the fecal samples at a solid-to-liquid ratio of 1:10 (*w*/*v*), followed by thorough homogenization. The mixture was centrifuged at 2000× *g* for 10 min. The resulting supernatant was filtered through gauze and then a 0.22 μm membrane filter to obtain the fecal microbiota transplantation (FMT) suspension. The FMT suspension was then centrifuged at 8000× *g* for 10 min and subsequently filtered through a 0.45 μm membrane filter to yield the FVT suspension. The FVT suspension was supplemented with 10% glycerol and stored at −80 °C. (Note: the physiological saline used for gavage in the CON group was also supplemented with 10% glycerol).

### 2.3. Diets and Compositional Analysis

Throughout the experiment, all birds received a corn–soybean meal-based basal diet. The formulation and nutritional composition of the basal diet are presented in Table 1. The diet was formulated to meet or exceed the nutrient requirements specified in the Nutrient Requirements for Yellow Feather Broilers (NY/T 3645-2020) (Ministry of Agriculture and Rural Affairs of China, 2020) [25].

### 2.4. Growth Performance

Recipient broilers were weighed at 8 and 21 days of age, following an 8 h fasting period prior to each weighing event. Feed intake was recorded daily on a per-replicate basis. The average daily gain (ADG), average daily feed intake (ADFI), and feed conversion ratio (FCR) were calculated using the collected data.

### 2.5. Immunological and Antioxidant

At 21 days of age, six recipient broilers were randomly selected per group (one bird per replicate) for sample collection. Approximately 5 mL of blood was collected from each bird via wing venipuncture into anticoagulant tubes, followed by centrifugation at 3500 r/min for 20 min at 4 °C. The resulting plasma supernatant was aliquoted and stored at −20 °C. Concurrently, liver and intestinal tissues (duodenum, jejunum, ileum) were homogenized in proprietary extraction buffer from commercial kits and stored at −20 °C for identical assays.

Plasma immunoglobulin levels, including those of Immunoglobulin A (IgA), Immunoglobulin G (IgG), and Immunoglobulin M (IgM), and cytokine concentrations, including those of Interleukin-1 beta (IL-1β), Interleukin-6 (IL-6), Interferon-gamma (IFN-γ), and Tumor Necrosis Factor-alpha (TNF-α), were determined using an enzyme-linked immunosorbent assay (ELISA) [26], while oxidative stress markers including malondialdehyde (MDA), superoxide dismutase (SOD), catalase (CAT), and glutathione peroxidase (GPX) were quantified through colorimetric methods [27]. Identical immunological and antioxidative parameter analyses were performed on liver and intestinal tissue homogenates. All procedures strictly followed the manufacturer’s protocols (Shanghai Enzyme-linked Biotechnology Co., Ltd., Shanghai, China).

### 2.6. Measurement of Intestinal Histomorphological Parameters

During systematic dissection, duodenal, jejunal, and ileal segments were excised from broilers for morphometric analysis. Intestinal length was measured using vernier calipers, with the intestinal index calculated as the ratio of the length (cm) to the body weight (g). Five-centimeter tissue sections from each intestinal segment underwent saline rinsing followed by fixation in 4% paraformaldehyde. Paraffin-embedded sections were prepared through standardized dehydration, clearing, and embedding procedures, with hematoxylin-eosin (HE) staining subsequently performed. For each sample, six intact villi and corresponding crypts were measured to determine villus height and crypt depth, with the villus height-to-crypt depth ratio (V/C) computed. Histological processing included the following: tissue trimming into 1-cm^2^ segments, vigorous saline rinsing, automated dehydration and paraffin infiltration, microtome sectioning at a 5 μm thickness, and staining via standardized hematoxylin–eosin protocols with xylene clearing (5 min) and neutral balsam mounting. Morphometric quantification was carried out utilizing light microscopy imaging with systematic randomization (five fields/slide) for software-assisted villus/crypt measurements [28].

### 2.7. RNA Extraction and qRT-PCR Analysis

Total RNA was isolated using TRIzol reagent (TransGen Biotech, Beijing, China), and its purity/concentration was spectrophotometrically quantified using a NanoDrop instrument (Thermo Fisher Scientific, Waltham, MA, USA). First-strand cDNA was synthesized with a reverse transcription kit (TransGen Biotech, Beijing, China). Subsequent qRT-PCR analysis was conducted on a Roche LightCycler 96 system with the following 20 µL reaction mixture: 15 µL PerfectStart Green qPCR SuperMix, 10 ng cDNA template, and 0.2 µM of each forward/reverse primer. Thermal cycling conditions comprised the following: initial denaturation at 95 °C for 30 s; 50 cycles of denaturation at 95 °C for 10 s, annealing at 60 °C for 15 s, and extension at 72 °C for 10 s. All reactions were performed in triplicate, with relative gene expression calculated via the 2^−∆∆Ct^ method [29]. Primer sequences for target genes are listed in Table 2, using β-actin as the endogenous reference gene.

### 2.8. Cecal Microbial Diversity

Cecal luminal samples obtained during systematic dissection underwent comprehensive microbial profiling. Genomic DNA extraction was performed via magnetic bead-based methodology using the TIANGEN DNA Isolation Kit (TIANGEN Biotech, Beijing, China). Subsequent procedures executed by Novogene Co., Ltd. (Beijing, China) included the following: 16S rRNA gene amplification targeting the V4 hypervariable region with primers 515F/806R and library construction employing the NEB Next^®^ Ultra™ II FS DNA Library Prep Kit (New England Biolabs, Ipswich, MA, USA) followed by paired-end sequencing (2 × 250 bp) on the Illumina NovaSeq 6000 platform (Illumina, San Diego, CA, USA). Bioinformatics processing implemented in QIIME2 encompassed raw read quality filtering (fastp), paired-end merging (FLASH) to generate Raw Tags, and ASV denoising with DADA2 [30]. The average number of high-quality sequences per sample was 98,566 reads (range: 51,334 to 143,797 reads). All samples were rarefied to 51,000 reads/sample prior to diversity metric calculation. Taxonomic assignment against the SILVA 138.1 database preceding downstream analyses on the Novogene Cloud Platform incorporated α-diversity indices (Shannon, Chao1, Simpson) and β-diversity metrics, and differential taxon identification was performed via an independent sample *t*-test, with all workflows conforming to standardized QIIME2 microbiome characterization protocols.

### 2.9. Statistical Analysis

Data were analyzed statistically using SPSS 26.0 (IBM Corp., Armonk, NY, USA). Continuous variables were assessed for normality with the Shapiro–Wilk test (α = 0.05). Normally distributed data underwent Levene’s test for homoscedasticity; those meeting homoscedasticity assumptions were analyzed by an independent samples *t*-test, while non-normal data were processed with Mann–Whitney *U* tests. Statistical significance is denoted as follows: * *p* < 0.05, ** *p* < 0.01, and “ns” = not significant (*p* ≥ 0.05).

## 3. Results

### 3.1. Growth Performance

**Figure 1:**
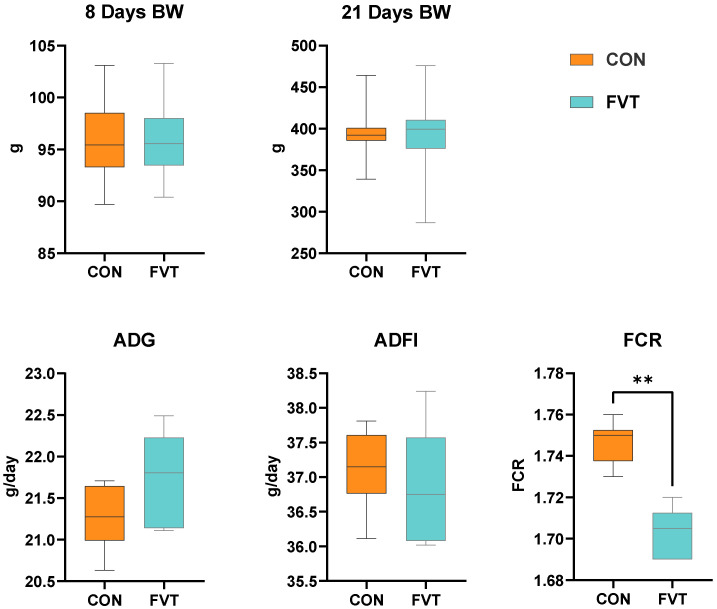
Effects of FVT on growth performance in broilers. Experimental groups: CON and FVT; growth performance: BW, ADG, ADF, and FCR. Statistical significance is denoted as follows: ** *p* < 0.01. The error bars are based on the standard error of means.

As shown in Figure 1, compared to the CON group, the FVT group exhibited no significant differences (*p* > 0.05) in initial body weight (8 Days BW, final body weight (21 Days BW), average daily gain (ADG), or average daily feed intake (ADFI). However, the FVT group demonstrated a highly significant reduction (*p <* 0.01) in the feed conversion ratio (FCR).

### 3.2. Blood Immunology and Antioxidation

**Figure 2:**
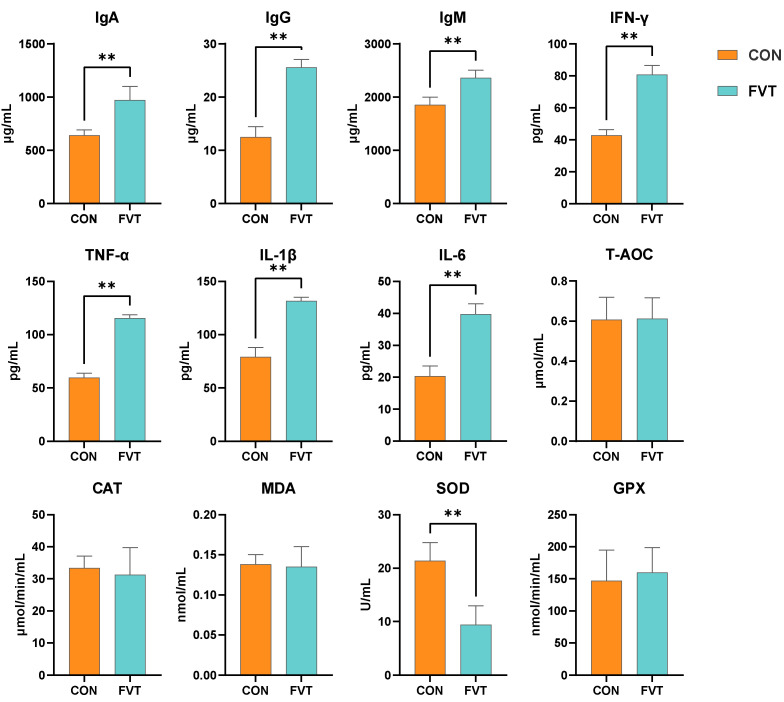
Effects of FVT on blood immunology and antioxidant capacity in broilers. Experimental groups: CON and FVT; immunological parameters: IgA, IgG, IgM, IFN-γ, TNF-α, IL-1β, and IL-6; oxidative markers: T-AOC, CAT, MDA, SOD, and GPX. Statistical significance is denoted as follows: ** *p* < 0.01. The error bars are based on the standard error of means.

Figure 2 demonstrates that for blood immune parameters, the FVT group exhibited highly significant increases (*p <* 0.01) in IgA, IgG, IgM, IFN-γ, TNF-α, IL-1β, and IL-6 compared to CON. Regarding blood antioxidants, the SOD value in the FVT group was highly significantly lower than that in the control group (*p <* 0.01).

### 3.3. Hepatic Immunology and Antioxidation

**Figure 3:**
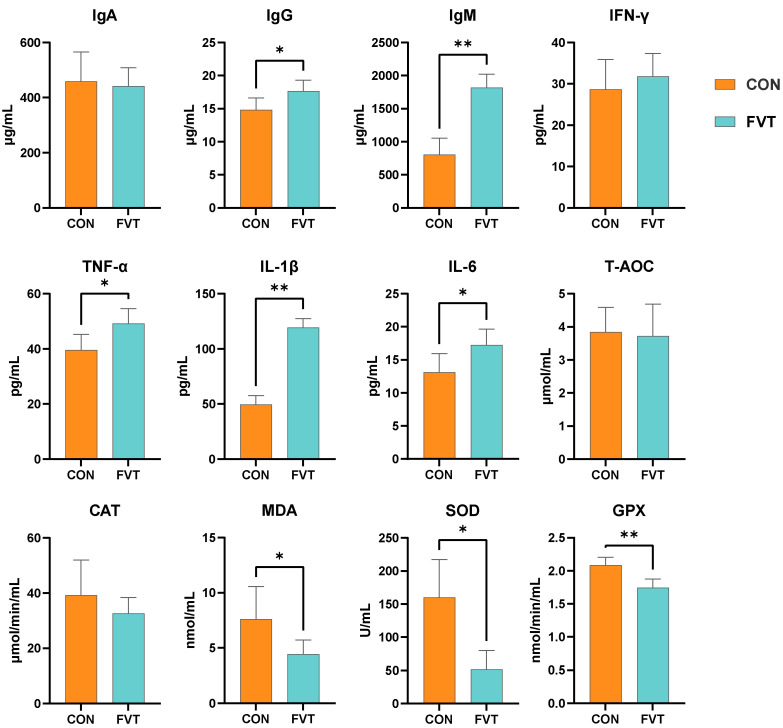
Effects of FVT on hepatic immunology and antioxidant capacity in broilers. Experimental groups: CON and FVT; immunological parameters: IgA, IgG, IgM, IFN-γ, TNF-α, IL-1β, and IL-6; oxidative markers: T-AOC, CAT, MDA, SOD, and GPX. Statistical significance is denoted as follows: * *p* < 0.05 and ** *p* < 0.01. The error bars are based on the standard error of means.

As presented in Figure 3, for the hepatic immune factors, IgG, TNF-α, and IL-6 were significantly elevated (*p <* 0.05) in FVT, whereas IgM and IL-1β showed highly significant increases (*p <* 0.01). For the hepatic antioxidants, the amounts of MDA and SOD were significantly reduced (*p <* 0.05), and the amount of GPX was highly significantly lower (*p <* 0.01) in FVT compared to CON. Additionally, histological examination revealed no abnormalities in liver sections from either group.

### 3.4. Intestinal Histomorphology

**Figure 4:**
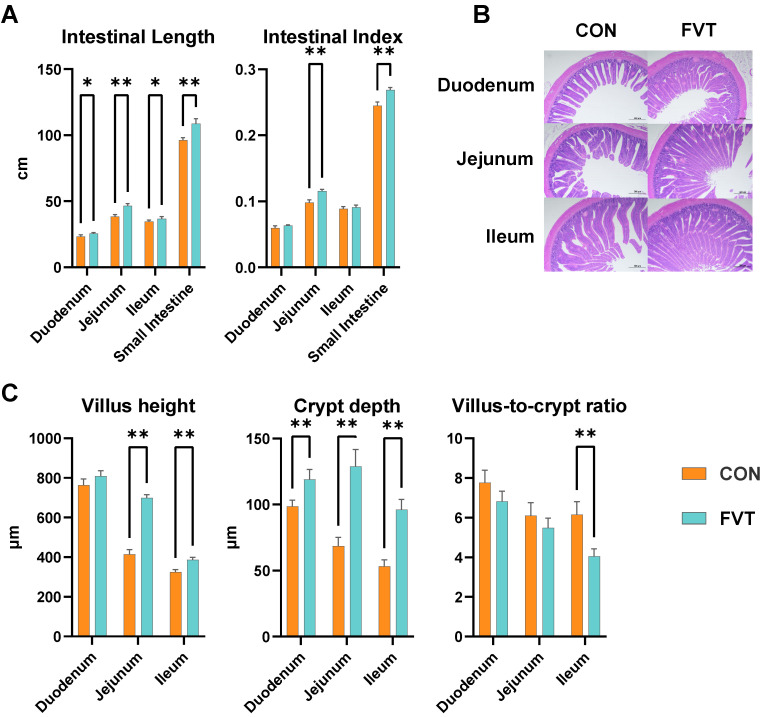
Effects of FVT on small intestinal histomorphology in broilers. (**A**) Small intestinal length and intestinal index. (**B**) Representative duodenal, jejunal, and ileal HE-stained sections. (**C**) Duodenal, jejunal, and ileal measurements of villus height, crypt depth, and V/C ratio. Statistical significance is denoted as follows: * *p* < 0.05 and ** *p* < 0.01. The error bars are based on the standard error of means.

As the primary site for nutrient absorption in broilers, the small intestine exhibited significant morphological alterations in the FVT group versus the CON group (Figure 4). Duodenal and ileal lengths were significantly elongated (*p* < 0.05), whereas jejunal and total intestinal lengths showed highly significant increases (*p* < 0.01). The jejunum index and total intestinal index were markedly elevated in FVT compared to CON (*p* < 0.01). Additionally, villus heights in the jejunum and ileum demonstrated profound enhancement (*p* < 0.01), crypt depths throughout duodenum, jejunum, and ileum were highly significantly deepened (*p* < 0.01), and the ileal villus-to-crypt ratio was substantially reduced in FVT relative to CON (*p* < 0.01).

### 3.5. Intestinal Immunology

**Figure 5:**
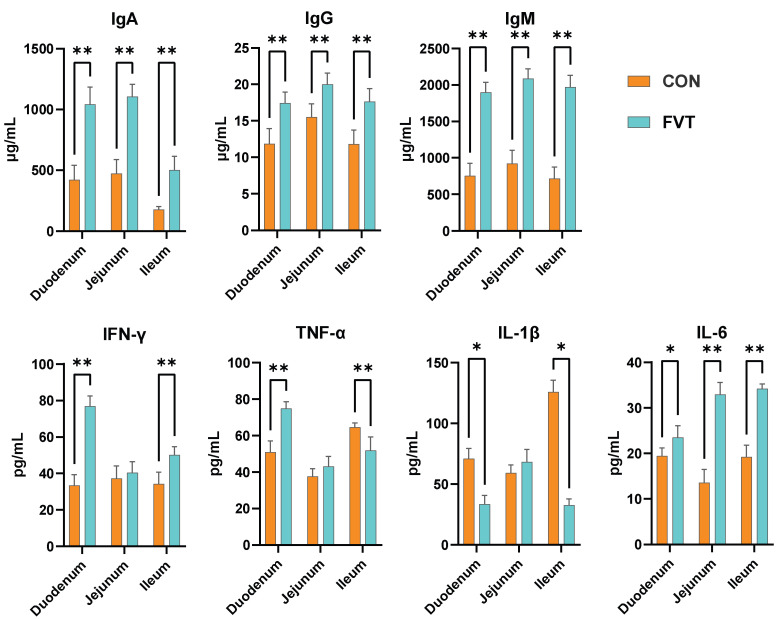
Effects of FVT on intestinal immunology in broilers. Experimental groups: CON and FVT; immunological parameters: IgA, IgG, IgM, IFN-γ, TNF-α, IL-1β, and IL-6. Statistical significance is denoted as follows: * *p* < 0.05 and ** *p* < 0.01. The error bars are based on the standard error of means.

Figure 5 indicates that immunoglobulin levels (IgA, IgG, IgM) across the duodenum, jejunum, and ileum were highly significantly elevated in the FVT group compared to those in the CON (*p* < 0.01). IFN-γ concentrations in the duodenum and jejunum similarly showed highly significant increases relative to those in the CON (*p* < 0.01). TNF-α displayed opposing regional effects: it was highly significantly increased in duodenum (*p* < 0.01) but highly significantly decreased in the ileum compared to in the CON (*p* < 0.01). IL-1β levels were significantly suppressed in the duodenum and ileum compared to those in the CON (*p* < 0.05). For IL-6, duodenal expression was significantly higher (*p* < 0.05), while jejunal and ileal levels exhibited highly significant upregulation relative to those in the CON (*p* < 0.01).

### 3.6. Expression of Genes Related to Intestinal Barrier and Nutrient Absorption

**Figure 6:**
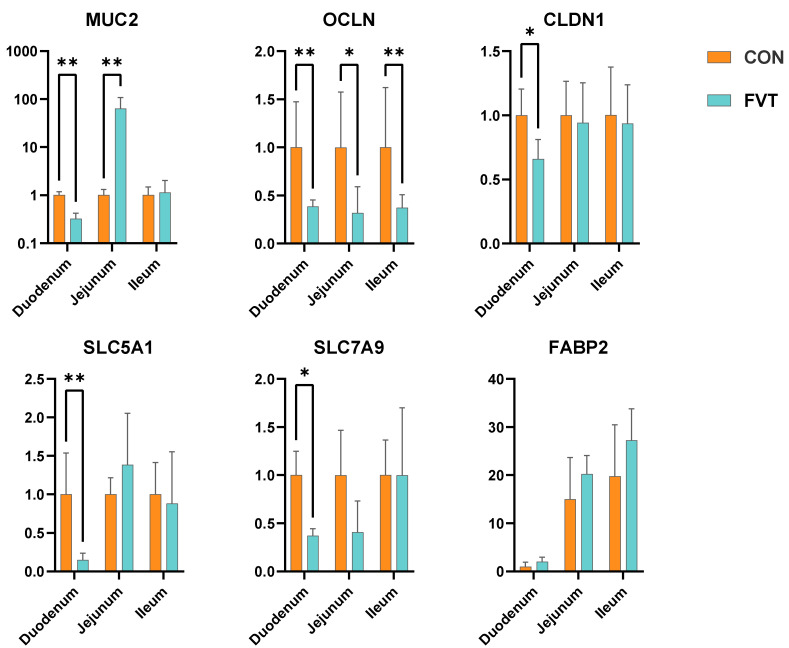
Effects of FVT on relative expression of genes related to intestinal barrier function and nutrient metabolism in broilers. Experimental groups: CON and FVT; intestinal barrier markers: *MUC2*, *OCLN* and *CLDN1*; nutrient transport markers: *SLC5A1*, *SLC7A9* and *FABP2*. Statistical significance is denoted as follows: * *p* < 0.05 and ** *p* < 0.01. The error bars are based on the standard error of means.

Figure 6 shows that in the FVT group, duodenal Mucin 2 (*MUC2*) and Occludin (*OCLN*) were highly significantly downregulated (*p <* 0.01), and Claudin 1 (*CLDN1*) was significantly downregulated (*p <* 0.05); jejunal *MUC2* showed a marked upregulation (*p <* 0.01) alongside *OCLN*’s significant decrease (*p <* 0.05); ileal *OCLN* was highly significantly downregulated (*p <* 0.01). For nutrient absorption genes, duodenal Solute Carrier Family 5 Member 1 (*SLC5A1*) and Solute Carrier Family 7 Member 9 (*SLC7A9*) were highly significantly and significantly decreased, respectively (*p <* 0.01 and *p <* 0.05).

### 3.7. Expression of Genes Related to Intestinal Development and Repair

**Figure 7:**
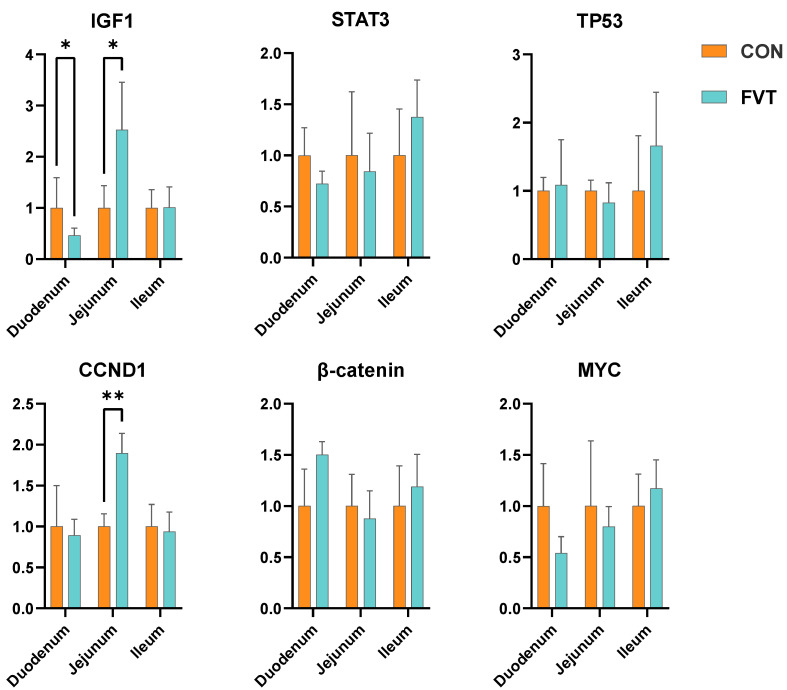
Effects of FVT on relative expression of genes related to intestinal development and repair in broilers. Experimental groups: CON and FVT; epithelial differentiation and apoptosis markers: *IGF-1*, *CCND1*, *STAT3*, *β-catenin*, *MYC* and *TP53*. Statistical significance is denoted as follows: * *p* < 0.05 and ** *p* < 0.01. The error bars are based on the standard error of means.

Figure 7 demonstrates the divergent regulation of growth factors in the FVT group: Duodenal Insulin-like Growth Factor 1 (*IGF-1*) exhibited significant suppression versus the CON group (*p* < 0.05), whereas jejunal *IGF-1* displayed elevated expression (*p* < 0.05) accompanied by *Cyclin D1* (*CCND1*)’s profound upregulation (*p* < 0.01).

### 3.8. Cecal Microbial Diversity

**Figure 8:**
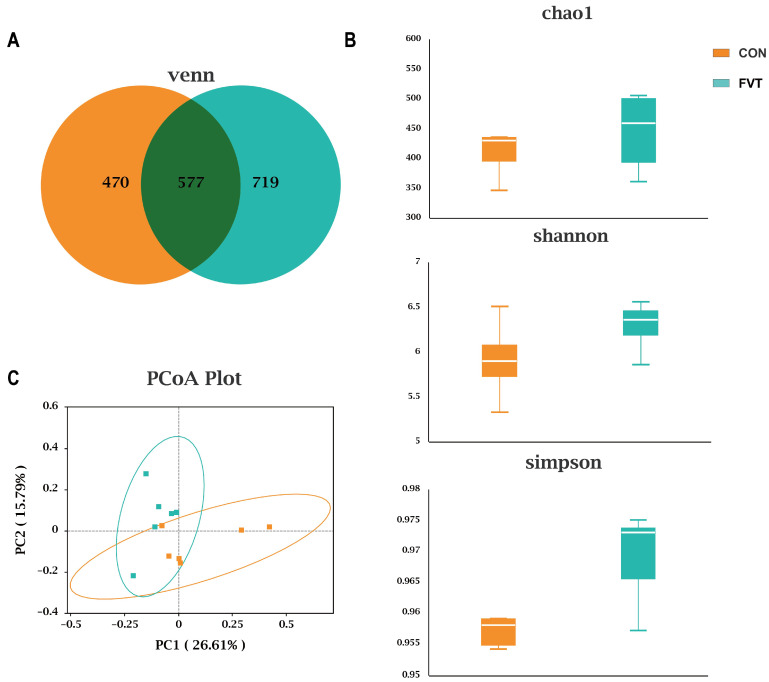
Effects of FVT on cecal microbial diversity in broilers. (**A**) Venn diagram of cecal ASVs. (**B**) α-diversity indices (Chao1, Shannon, and Simpson). (**C**) PCoA plot of β-diversity (Bray–Curtis distance).

Figure 8A shows that CON and FVT shared 577 ASVs, with the FVT group exhibiting more unique ASVs (249 vs. CON). Figure 8B indicates no significant differences in α-diversity indices (Chao1, Shannon, Simpson; *p >* 0.05). Figure 8C reveals distinct microbial community clustering by PCoA, with Adonis testing confirming highly significant β-diversity differences (*p <* 0.01), indicating divergent community structures.

**Figure 9:**
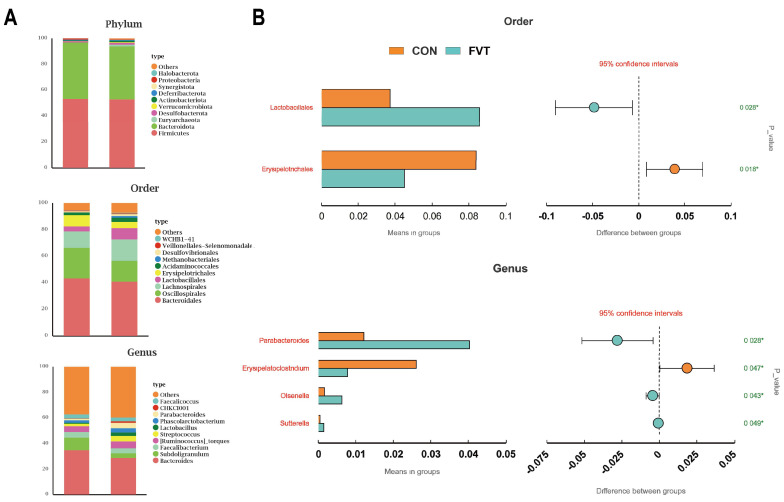
Effects of FVT on cecal microbial composition in broilers. (**A**) Taxonomic composition at phylum, order, and genus levels. (**B**) Differential taxa at order and genus levels identified by *t*-test. Statistical significance is denoted as follows: * *p* < 0.05.

Figure 9A shows that FVT altered microbial composition at the phylum, order, and genus levels. Figure 9B demonstrates that at the order level, the FVT group had significantly higher relative abundance of *Lactobacillales* and lower relative abundance of *Erysipelotrichales* than the CON group (*p <* 0.05); at the genus level, the FVT group had significantly higher *Parabacteroides*, *Olsenella*, and *Sutterella* counts, but lower *Erysipeloclostridium* counts (*p <* 0.05).

## 4. Discussion

Microbial transplantation from dominant populations has been demonstrated to improve corresponding phenotypes in recipient animals. Ma et al. showed that transplanting the gut microbiota from high-body-weight healthy chickens to chicks significantly enhanced growth performance and intestinal development [14], effects typically attributed to gut bacteria. Building upon FMT, this study employed a 0.45 μm membrane filter to remove the majority of bacterial components, thereby transplanting the gut virome and metabolites from healthy high-body-weight chickens to chicks via FVT. We observed a highly significant decrease in FCR (*p <* 0.01) without significant differences in body weight compared to those in the CON group, indicating improved feed utilization in FVT-treated chicks. Combined with the documented probiotic effects of bacteriophages in animal studies, these findings suggest that the gut virome and metabolites may contribute to the growth-promoting effects of FMT [17,18].

Immune and antioxidant parameters serve as key indicators of systemic health status. Pro-inflammatory cytokines (TNF-α, IL-1β, IL-6, IFN-γ) are critical mediators of inflammatory immune responses [31,32]. Immunoglobulins constitute the frontline of humoral immunity: IgM acts as the first responder, while IgA serves as the dominant effector in mucosal immunity, blocking pathogen adhesion [33]. IgG neutralizes pathogens and activates the complement system to enhance host defense [34]. Antioxidant homeostasis is essential for counteracting oxidative damage; SOD, CAT, and GPX represent core antioxidant enzymes that mitigate peroxidation, whereas T-AOC and MDA serve as comprehensive assessment markers and damage indicators, respectively, directly reflecting systemic oxidative stress levels [35,36].

Assessments of immune parameters in broiler blood and liver revealed that compared to the CON, FVT significantly upregulated (*p <* 0.05) immune factors and immunoglobulins (IFN-γ, TNF-α, IL-1β, IL-6, IgA, IgG, IgM) in both blood and liver, indicating systemic immune activation in FVT-treated broilers. This suggests that FVT may trigger host immune responses through undefined mechanisms, potentially contributing to adverse effects. Regarding antioxidation, blood SOD and hepatic GPX were highly significantly lower (*p <* 0.01) in FVT, while hepatic MDA and SOD were significantly lower (*p <* 0.05). The reduction in hepatic MDA indicates decreased oxidative products; combined with the decrease in SOD and GPX, this suggests that the decline in antioxidant system activity may result from negative feedback regulation due to reduced oxidative reactions in the body. Host immune and antioxidant indicators correlate with gut microbial structure. Microbes can influence systemic health through metabolites via the gut–liver axis [37]. Critically, harmful viruses and virulence factors compromise the host intestinal barrier, thereby increasing intestinal permeability [38]. This enhanced permeability allows for greater permeation of microbes and their metabolites into the bloodstream, subsequently triggering hepatic and systemic changes in immune and antioxidant systems via the gut–liver axis [39], which suggests that intestinal barrier damage in FVT-treated broilers may be one contributing factor to the observed immune dysregulation and antioxidant alterations in this trial.

Improved feed utilization indicates that FVT may enhance digestive system development in broilers. As the primary site for nutrient absorption, the small intestine was significantly greater in length in all three segments, as well as showing a deeper crypt depth and significantly increased villus height in the jejunum and ileum in the FVT group compared to the CON. However, only the jejunal index showed significant elevation, indicating that FVT improved intestinal histomorphology with the most pronounced pro-developmental effects observed in the jejunum. Subsequent analysis of intestinal immunology revealed highly significant upregulation of immunoglobulins across all segments in the FVT group. Unlike in the duodenum and ileum, jejunal IFN-γ, TNF-α, and IL-1β showed no significant differences from the CON, with only IL-6 being highly significantly elevated. This demonstrates that while FVT induced intestinal immune dysregulation, its adverse impact on the jejunum was comparatively minor.

The integrity of the intestinal barrier directly determines the body’s nutrient absorption and immune function status [40]. Among these, the physical barrier (including intestinal epithelial cells, tight junction proteins, mucins, etc.) [41,42] is a key element in maintaining barrier integrity. Given the critical regulatory role of gut microbiota and their metabolites on the barrier [43], combined with intestinal morphological changes observed in this experiment, this study conducted quantitative analysis of the following intestinal barrier- and nutrient metabolism-related genes: tight junction proteins *OCLN* and *CLDN1*, along with goblet cell-secreted *MUC2*, maintain intestinal barrier integrity [44], while *SLC5A*1 (sodium/glucose cotransporter), *SLC7A9* (amino acid transporter), and Fatty Acid-Binding Protein 2 (*FABP2*: fatty acid-binding protein) mediate key nutrient absorption pathways [45,46,47]. Assessment of barrier and nutrient absorption genes showed significant downregulation of *MUC2*, *OCLN*, and *CLDN1* in the duodenum (*p <* 0.05) and highly significant downregulation of *OCLN* in the ileum. Notably, despite significant downregulation of key duodenal nutrient absorption genes (SLC5A1, SLC7A9; *p* < 0.05), feed utilization improved. This apparent contradiction may arise from the following: (1) compensatory increases in structural metrics (intestinal length, crypt depth), and (2) potential functional adaptations, which might include alterations in intestinal permeability or other compensatory transport mechanisms, facilitating nutrient uptake despite the reduced expression of specific transporters. Conversely, the jejunum exhibited highly significant upregulation of *MUC2* alongside a significant downregulation of *OCLN* (*p <* 0.05). Collectively, these results indicate less severe barrier impairment in the jejunum, with significant enhancements of physical barrier function, minimal variations in immune factors, and substantial structural improvement. This aligns with previous findings demonstrating that FMT and FVT promote broiler intestinal development [14,15,23,24,48], suggesting that jejunal improvement likely contributes to enhanced feed utilization. These observations further substantiate the virome and metabolites as functional components mediating FMT’s beneficial effects and highlight the spatial heterogeneity and dual nature of FVT’s impact on intestinal development.

To further elucidate intestinal development mechanisms, repair-related genes were examined. *IGF-1* and *CCND1* promote epithelial differentiation and suppress apoptosis (with *CCND1* being *IGF-1*-inducible) [49,50], Signal Transducer and Activator of Transcription 3 (*STAT3*) mitigates excessive inflammation damage [51], Beta-Catenin (*β-catenin*: Wnt pathway) collaborates with MYC Proto-Oncogene (*MYC*) to drive cell cycle progression [52], and Tumor Protein P53 (*TP53*) is crucial for cellular repair [53]. Results showed a significant downregulation of duodenal *IGF-1* (*p <* 0.05) in FVT with no significant changes in other genes, indicating suppressed duodenal cell proliferation consistent with observed barrier impairment and metabolic gene downregulation. Conversely, jejunal *IGF-1* was significantly upregulated (*p <* 0.05), corroborating prior FMT research [15], and *CCND1* was highly significantly upregulated, suggesting activation of the *IGF-1*/*CCND1* pathway. Combined with the promotion of jejunal development and barrier enhancement, this indicates that FVT stimulates jejunal epithelial cell proliferation and differentiation. This likely explains the optimal pro-developmental effect of FVT in the jejunum and further underscores the spatial heterogeneity and dual nature of FVT’s intestinal effects.

Finally, cecal 16S diversity analysis revealed that FVT enhanced microbial diversity in chicks, a factor generally beneficial for early growth. Compositionally, the FVT group showed a significantly higher (*p <* 0.05) abundance of *Lactobacillales* and significantly lower (*p <* 0.05) *Erysipelotrichales* counts compared to the CON group. *Lactobacillales*, identified as a primary functional order in previous FMT studies, exhibits well-documented probiotic properties [54,55], while *Erysipelotrichales* is considered an opportunistic pathogenic order [56]. The improved microbial community structure induced by FVT could be one of the reasons contributing to the enhanced growth performance in broilers of the FVT group, while this improvement may result from the targeted lytic activity of bacteriophages or promotion via metabolic substrates [57,58], indicating that the virome and metabolite profile of high-growth-performance chickens possesses probiotic potential. However, the potential pathogenic risks associated with FVT warrant consideration.

In conclusion, FVT from high-body-weight chickens to chicks exhibits a dual nature. On one hand, it improves feed utilization and promotes intestinal development; on the other, it induces abnormalities in immune and antioxidant parameters, impairs intestinal barrier function in certain segments, and demonstrates spatial heterogeneity in its intestinal effects. Specifically, while FVT promoted jejunal development and barrier integrity—consistent with prior FMT and FVT research—it caused significant barrier impairment in the duodenum. This coexistence of benefits and drawbacks highlights the need to identify the principal functional components within FMT or FVT. Developing safe, targeted artificial formulations based on these components represents a crucial future direction for microbial interventions.

## 5. Conclusions

This study confirms that fecal virome transplantation (FVT) from healthy high-body-weight yellow-feathered broilers to chicks significantly improves feed utilization and promotes intestinal development, demonstrating that the virome and metabolites are key functional components mediating the growth-enhancing effects of FMT. Differential responses across intestinal segments reveal spatial heterogeneity in FVT-induced intestinal development, while immune dysregulation and impaired barrier integrity highlight the dual nature of its effects. The underlying mechanisms require further elucidation. Notably, the limitations of this study include the following: the specific composition of transplanted virome and metabolites was not characterized; protein-level validation (e.g., Western blot) of intestinal barrier and nutrient metabolism-related genes was not performed; and the gut–liver axis mechanism requires exploration. Given the significant growth-promoting potential of FMT/FVT, we propose that identifying their primary functional components and developing safe, targeted artificial formulations represent a critical future direction for microbial-based interventions.

## Figures and Tables

**Table 1:** Composition and nutrient levels of basal diets (%, dry matter basis).

Item	Content
Ingredients, %	
Maize	40.85
Soybean meal	43.35
Soybean oil	9.05
Premix ^1^	5.00
L-Lysine HCl	0.10
DL-Methionine	0.20
Calcium carbonate	0.30
Calcium hydrogen phosphate	1.15
Total	100
Nutrient levels ^2^	
Crude protein, %	21.51
Crude fat, %	11.22
Crude ash, %	6.97
ME, MJ/kg	12.38
Lysine, %	1.29
Methionine, %	0.50
Calcium, %	1.02
Total phosphorus, %	0.74

^1^ Supplying per kilogram feed: 10,000 IU vitamin A, 10,000 IU vitamin D3, 4 mg vitamin E, 0.75 mg vitamin K, 0.7 mg thiamine, 1.8 mg riboflavin, 0.55 mg pyridoxine, 0.01 mg vitamin B12, 11.25 mg niacin, 3.5 mg pantothenic acid, 0.75 mg folic acid, 0.05 mg biotin, 5 g NaCl, 4.25 g Ca, 1.125 g P, 75 mg Zn, 100 mg Mn, 125 mg Fe, 15 mg Cu, 0.75 mg I, 0.3 mg Se, 0.25 g Lysine. ^2^ ME values were calculated from data provided by NY/T 3645-2020, while the others were measured values.

**Table 2:** Genes and primer sequences used for qRT-PCR analysis.

Gene	Primer Sequences (5′ to 3′)	Accession No.
*β-actin*	F_ATTGTCCACCGCAAATGCTTC R_AAATAAAGCCATGCCAATCTCGTC	NM_205518.2
*MUC2*	F_TTCATGATGCCTGCTCTTGTG R_CCTGAGCCTTGGTACATTCTTGT	XM_040673064.2
*OCLN*	F_GGCGGAGGGCCACCA R_GTCGTCCACGTAGTAGGAGC	NM_205128.1
*CLDN1*	F_TACTCCTGGGTCTGGTTGGT R_GTGCTGACAGACCTGCAATG	NM_001013611.2
*SLC5A1*	F_TCCACCGCCATAAGGATCAA R_GGTTGGTTGAGTACATAGCCCAT	NM_001293240.2
*SLC7A9*	F_TGGCACCAATTATCACCGCA R_GTGCATAGTGATGGGCTCTGAT	XM_046925531.1
*FABP2*	F_TCATGGAAGCAATGGGCGTG R_TTCGATGTCGATGGTACGGAAG	NM_001007923.2
*IGF-1*	F_TGCCAAAAGCACAAAAGGAAGT R_AAGTACCCTGCAGATGGCAC	NM_001004384.3
*STAT3*	F_CTCAGGTAGTGCTGCTCCGT R_GGGCAGGTCAATGGTATTGC	XM_040653164.2
*TP53*	F_CGCCGTGGCCGTCTATAA R_GTACAGTCAGAGCCCACCTCG	NM_205264.1
*CCND1*	F_ACCCGACGAGTTACTGCAAA R_TAGCGCACAGAGCCACAAAA	NM_001396513.1
*β-catenin*	F_ATTGTCCACCGCAAATGCTTC R_AAATAAAGCCATGCCAATCTCGTC	NM_205518.2
*MYC*	F_CAGCAGCGACTCGGAAGAAGAA R_AACTTTAGCCTCTTGGCGGC	NM_001030952.2

## Data Availability

The original contributions presented in this study are included in the article. Further inquiries can be directed to the corresponding authors.
